# Genomic perplexity and the evolution of context-dependent function

**DOI:** 10.1093/molbev/msag041

**Published:** 2026-02-25

**Authors:** James O McInerney

**Affiliations:** Department of Evolution, Ecology and Behaviour, Institute of Infection, Veterinary, and Ecological Sciences, Biosciences Building, University of Liverpool, Crown Street, Liverpool L69 7ZB, UK

**Keywords:** genomic perplexity, horizontal gene transfer, mutation, information theory, machine learning

## Abstract

The fundamental principle that selection acts on a gene's function often assumes implicitly that this function is fixed and intrinsic. However, empirical evidence from pangenomics, synthetic biology, and GWAS consistently demonstrates that organismal function is highly context-dependent, varying across genomic backgrounds and cellular states, even for core genes. Drawing a conceptual parallel with modern large language models (LLMs), I propose that genomes, like LLMs, do not encode fixed functions but rather “probability distributions” over functional and phenotypic outcomes. This framework draws a conceptual analogy between epistasis and transformer-style “attention mechanisms,” suggesting that genomic context weights the influence of distant genetic elements. I also introduce the concept of “genomic perplexity”—an information-theoretic measure of the statistical unexpectedness and incompatibility of a genetic element within its host context. I demonstrate how perplexity serves as a quantifiable metric for the well-known fitness cost associated with interspecies gene flow (eg horizontal gene transfer (HGT) and introgression), where a new gene represents a high-perplexity token. This perspective formalizes long-standing observations of genomic fit and provides a testable framework for predicting the integration potential of accessory genes and directing future research in synthetic biology and evolutionary modeling.

## Introduction

Natural selection acts on the fitness consequences that genes produce across diverse contexts, not on their biochemical activities directly but on how those activities contribute to survival and reproduction in each genomic and environmental setting. While the modern synthesis established selection as the primary force shaping genomic content (Ciccarelli et al. 2006; Douglas and Shapiro 2024; Goyal 2018; Messier and Stewart 1997; O'Connell and McInerney 2005; van Opijnen et al. 2015), and despite recognition of epistasis and environmental effects, selection is typically discussed as acting on fixed gene functions. However, function itself only emerges through interaction. For example, the magnesium transporter SP_0185 in *Streptococcus pneumoniae* performs the biochemical activity of magnesium acquisition in all strains. This gene is completely essential for growth in some strains and completely dispensable in others, depending on whether a redundant transporter is present (Rosconi et al. 2022). SP_0185 does not “have the function” of being essential or dispensable; its fitness contribution emerges from its interaction with the rest of the genome. The *lac* operon, which is normally considered to function in the metabolism of lactose (Jacob and Monod 1961), can also function in the breakdown of X-gal, in the absence of lactose. Therefore, in differing environmental situations, the *lac* operon has different functions. The distinction drawn here is not that molecular activities vary unpredictably, rather that the fitness consequences of those activities depend on genomic and environmental context. The distinction between innate function residing in a genetic element, and the emergence of function in context, has practical consequences, and indeed it fundamentally changes how we model and predict biological systems.

It is important to distinguish between biochemical function, which is the molecular activity of a gene product, and what I term here as organismal or fitness function, defined as the role that such an activity plays in survival and reproduction. For example, an esterase retains its ability to hydrolyze ester bonds across vastly different genomic contexts. Its biochemical specificity is largely context independent. However, whether the possession of an esterase is beneficial, neutral, or harmful depends entirely on what other enzymes are present, what substrates are available, and what metabolic demands the organism faces. Throughout this perspective, “function” refers to the fitness consequences of genomic component activity rather than the biochemical activity itself.

In prokaryotic pangenomes the same gene family can appear in radically different genomic contexts across strains (Beavan et al. 2024; McInerney et al. 2017). It is worth noting that gene families, by virtue of containing paralogous members with divergent sequences and expression patterns, may exhibit broader functional distributions than individual genes; however, even a single gene can generate context-dependent fitness outcomes when its molecular activity interacts differently with varying genomic backgrounds or environmental conditions. When we observe that a gene family is present in some genomes but absent from others or that it participates in different pathways depending on which other genes are present, we are not seeing a gene with multiple functions or variable importance. We are seeing that the function itself is not a property of genes but an “emergent outcome” of genes operating within specific contexts. The phenylacetate degradation gene cluster exemplifies this point, given that its component genes are strongly conserved across bacterial lineages (Martin and McInerney 2009), even though individual genes demonstrate remarkable functional breadth. *PaaK*, for example, activates diverse substrates from fatty acids to antibiotic precursors depending on what molecules are available and what other enzymes are present (Jiao et al. 2022; Law and Boulanger 2011). A gene that produces essential metabolic products in one environment but is redundant in a different condition will be selected based on the integrated fitness effects across both contexts, weighted by their frequency and importance (Domingo-Sananes and McInerney 2021). This is why the same gene can be essential in one strain and dispensable in another (Beavan and McInerney 2022; Rosconi et al. 2022): not because the gene's function has changed but because function only exists through context. These genes persist throughout evolution not because they encode fixed functions but because they reliably generate adaptive outcomes across the diversity of contexts encountered by their host organisms.

The neutrality-selection debate, initiated by Lewontin and Hubby's allozyme studies and formalized by Kimura and Ohta, questioned whether genetic variation was selectively neutral (Kimura 1983; Lewontin and Hubby 1966; Ohta 1973). Initial observations showed excessive variation incompatible with selection-only models, leading to the neutral theory. Modern models recognize the simultaneous existence of positively selected, negatively selected, and neutral sites within single genes (Yang 1998). Context-dependency adds another dimension, with various genetic elements being neutral in one genomic or environmental context but under strong selection in another, creating a high-dimensional, dynamic fitness landscape where selective consequences can shift with each change in background or environment.

That gene function varies with context is a principle long recognized through studies of epistasis, norm of reaction, and gene-by-environment interactions (Des Marais et al. 2013; Wolf 2000). Rather than viewing context-dependency as complicating “true” gene function, I propose that selection acts on probability distributions that genes generate across contexts, not on the singular functions of genetic components. Drawing parallels with transformer models in natural language processing, which succeed by using context-dependent rather than fixed meanings (Vaswani et al. 2017), I propose that evolution shapes genomes not to encode fixed functions but to optimize these probability distributions across actually encountered contexts. This framework generates testable predictions about horizontal gene transfer and introgression success (through perplexity metrics), pangenome structure (through context-frequency distributions), and why certain genes persist despite apparent redundancy (through their contribution to adaptive probability distributions).

## The genome as a context-dependent system

For decades, natural language processing attempted, and largely failed, to understand language through fixed word definitions and explicit grammatical rules (Panchendrarajan and Zubiaga 2024; Wermter et al. 1996). Meaning was treated as an intrinsic property of each word, and these meanings were encoded in dictionaries and linguistic structures. This approach faltered for a combination of reasons, such as the fact that context always matters, ambiguity is endemic, and the same word means different things in different contexts. The breakthrough came with a conceptual change, where instead of assigning fixed meanings, researchers began treating words as high-dimensional vectors whose meaning emerged from patterns of co-occurrence and positional relationship (Harris 1954; Mikolov et al. 2013). A word is understood not through its definition but through its position in a vast relational network (Turney and Pantel 2010). In modern transformer architectures (Vaswani et al. 2017), this is made explicit. Words do not carry predefined meanings, but instead, the model learns probability distributions over meaningful outputs given observed context. The analogy to genes is reasonably straightforward. A word's meaning emerges from its relationship to surrounding and co-occurring words, and in an analogous way, a gene's function emerges from its relationship to surrounding genomic, cellular, and environmental context. The question “what does this gene do?” can be just as context-dependent as “what does this word mean?”. A word's contribution to a sentence's meaning depends on surrounding words while its phonetic form remains constant; a gene's contribution to fitness depends on genomic context while its biochemical activity remains largely fixed.

In transformer language models, an attention mechanism allows each word to directly influence prediction based on every other word in the sequence (Vaswani et al. 2017). The model learns which words are relevant to predicting the next token, and the model weights are the outcome of this learned importance. A word's contribution to the final prediction depends on how much “attention” it receives from other words in context (Vaswani et al. 2017). This maps directly onto epistasis in genetics. While attention weights in language models are computational constructs that do not necessarily correlate directly with causal importance (Jain and Wallace 2019), the analogy remains useful as a conceptual frame for understanding how genetic elements exert context-dependent influence. The phenotypic effect of a focal allele depends on the genetic background, and specifically on the weighted influence of other loci. A mutation's effect, or the effect of an acquired or lost gene, depends on which other genetic variants are present and their state (eg their expression or their actual sequence). In the same way that attention weights reflect the learned reality that word meanings depend on context, epistatic interactions demonstrate the evolutionary reality that mutation effects depend on genetic background. The key insight is that both systems perform their function “through” relational weighting. Meaning in language models emerges from attention weights, while function in genomes emerges from the network of epistatic, regulatory, metabolic, and environmental interactions that weight each element's contribution. Neither is predetermined; both are learned (or evolved) through interaction with training data (or selective pressures).

Generative language models do not output a single “correct” word. They output probability distributions over possible next tokens (Bengio et al. 2003; Brown et al. 2020). Given context, the model computes the likelihood of each possible continuation. The most probable token is usually selected, but less probable tokens remain possible, and this stochasticity is an important feature of the model. Allowing the stochastic use of slightly sub-optimal words enables the model to generate diverse outputs and to handle genuine ambiguity in language.

Biological systems work similarly. Gene expression is fundamentally stochastic, with identical cells in identical conditions showing substantial variation in expression levels, protein abundance, and phenotypic outcomes (Elowitz et al. 2002; Raj and van Oudenaarden 2008). Regulatory networks have evolved the capacity to generate this stochasticity, and this enables bet-hedging strategies (Veening et al. 2008) and phenotypic heterogeneity even within clonal populations. Evolution tunes these probability distributions through parameters like promoter strength, transcription factor availability, and regulatory architecture. Natural selection has not resulted in deterministic outcomes; rather, it has led to genomes generating adaptive phenotypes with appropriate frequency across the contexts they encounter. A regulatory network producing only fixed outputs would be fragile and inflexible, while one generating a distribution of outcomes is more robust and evolvable.

Crystallins, for example, demonstrate how structural attention can be completely reorganized throughout evolutionary history. The δ-crystallin in some bird eye lenses is a recruited argininosuccinate lyase (Piatigorsky et al. 1994). In liver cells, the catalytic residues have maximum structural attention, because their identity, or character-state, entirely determines enzymatic function. But when the encoding gene is expressed solely in the lens, these same residues have near-zero attention for the relevant function (optical properties). Variation at the catalytic positions doesn't affect transparency or refractive index, so selection no longer constrains the evolution of these amino acids. The probability distribution of functional outcomes shifted from 100% enzymatic in metabolic contexts to 100% structural in optical contexts.

This shift in attention patterns extends to environmental contexts. Lysozyme in most mammals functions at neutral pH, but ruminant stomach lysozyme has evolved to function at pH 2 to 4 (Messier and Stewart 1997), with different amino acid residues having different attention weightings in the different environments.

Comparable reweighting of functional attention also occurs in multicellular regulation. For instance, alternative splicing, enhancer modularity, and phenotypic plasticity all demonstrate how eukaryotic systems evolve to manage context-dependent distributions of outcomes. In alternative splicing, a single gene can generate a range of protein isoforms depending on which exons are included, and this inclusion pattern is governed by cell-type-specific RNA-binding proteins and chromatin state. Natural selection therefore acts not only on the fitness effects of any single isoform but also on the distribution of isoforms produced across developmental and environmental contexts. Retaining multiple exons and regulatory motifs enables organisms to shift expression probabilities adaptively, thereby producing different functional outcomes in different tissues or conditions, while natural selection maintains this diversity when the aggregate fitness benefit of plastic, context-responsive expression outweighs the cost of maintaining unused variants.

The principles of context-dependent function extend naturally to developmental systems, where identical genetic material generates distinct phenotypic outcomes across time and cell type. Developmental gene regulatory networks represent an extreme case of shifting context, where the same transcription factor may act as an activator in one cell type and a repressor in another, depending on chromatin state, cofactor availability, and network topology (Davidson and Erwin 2006). Pioneer transcription factors exemplify “regulatory perplexity,” in action (the statistical unexpectedness of regulatory elements in their genomic context—formalized in the third section, Perplexity, fitness cost, and gene acquisition), by engaging previously inaccessible chromatin, they introduce unfamiliar regulatory logic that can reconfigure developmental trajectories (Iwafuchi-Doi and Zaret 2014; Zaret and Carroll 2011). Cell fate decisions emerge from transitions through regulatory state spaces rather than deterministic gene functions (Graf and Enver 2009; Trapnell et al. 2014). While a comprehensive treatment of developmental context-dependency is beyond the scope of this perspective, the perplexity framework presented in the third section below provides a natural quantitative language for these phenomena.

Context-dependency becomes particularly clear when we extend the language model analogy to pangenomes. A pangenome is the complete set of genes found across all strains of a species, partitioned into core genes (present in all or nearly all strains) and accessory genes (present in some but not all strains). In the same way that a language model learns probability distributions over word sequences by exposure to diverse texts representing different authors, genres, and contexts, genomes encode probability distributions over functional outcomes shaped by evolution across diverse genomic and environmental contexts. A pangenome can similarly be viewed as a distribution of functional modules across multiple genomic contexts. When we observe that a gene produces function differently in different strains, we are witnessing the natural equivalent of how a word embedding in a language model depends on its token context—the same element can have a different function depending on what surrounds it.

Core genes represent the most evolutionarily constrained probability distributions, producing consistent outcomes across most contexts through strong purifying selection. Accessory genes reveal the long tail of that distribution: functional outcomes that are locally adaptive in some contexts but disadvantageous, or neutral in others (Domingo-Sananes and McInerney 2021). Pangenomes therefore represent the realized diversity of genomic contexts that natural selection has had to optimize across, and in doing so, pangenomes display the fundamental principle that the function of a genetic element is not determined by its sequence alone; rather, it emerges from the interplay between sequence, genomic context, and environmental circumstance (McInerney 2023), which aligns with prior work on evolutionary assembly patterns of prokaryotic genomes (Press et al. 2016), chromosomal organization of horizontal gene transfer (Oliveira et al. 2017), and core-genome disharmony in recombining bacteria (Taylor et al. 2024; Townsend et al. 2003). This language model framework does not replace existing evolutionary theory but offers a complementary lens for understanding patterns that have long been observed.

## Perplexity, fitness cost, and gene acquisition

The perplexity metric quantifies how “surprised” a model is by an observed sequence (Jelinek 1977; Shannon 1948). Calculated as $2^{\left(cross\;entropy\right)}$, it measures how many bits of information are needed to encode an observed sequence given a model's learned probability distribution. A language model trained on English literature has low perplexity for typical English sentences, moderate perplexity for technical papers, and high perplexity for random character sequences.

When a gene is acquired through horizontal gene transfer (HGT) or introgression, it typically arrives in a genomic context differing from where it evolved (Beavan et al. 2024; Hall et al. 2020; McInerney et al. 2020; Sela et al. 2021). The recipient genome has evolved specific statistical structures, including codon preferences, regulatory syntax, metabolic pathway organization, and chromosomal architecture, that a transferred gene may violate (Callens et al. 2021). A well-integrated native gene has low perplexity relative to the genome's evolved patterns, while a transferred gene initially has high perplexity because it is statistically unexpected, creating fitness consequences both negative and positive (Callens et al. 2021).

When mammalian lysozyme first encountered acidic stomach environments, it likely had high perplexity because the protein was optimized for neutral pH. Evolution would have reduced this perplexity by shifting attention weights, which is to say that residues important at neutral pH became variable, while previously unimportant residues came under strong selection. Similarly, metabolic enzymes recruited as crystallins would have initially carried attention patterns optimized for catalysis. Evolutionary processes would have shifted structural attention from catalytic residues to surface residues affecting solubility and light transmission. Conservation represents high attention weights maintained across diverse contexts, while positive selection reflects attention weights shifting as contexts change.

Genomic perplexity can be calculated as the combined statistical unexpectedness across multiple molecular dimensions. Table 1 presents the mathematical framework for each perplexity dimension and its integration into total genomic perplexity.

**Table 1:** Mathematical framework for genomic perplexity.

Perplexity dimension	Equation and description
Codon usage	$PP_{\mathrm{codon}}=2^{-\frac{1}{N}\sum \log\;2P(\mathrm{codo}n_{i}|\mathrm{recipient})}$
	Measures statistical unexpectedness of synonymous codon usage patterns. High perplexity indicates rare codon usage causing slow translation and tRNA depletion. *N* = codons in transferred gene; $P(\mathrm{codo}n_{i}|\mathrm{recipient})$ = codon probability in recipient
Protein structure	$PP_{\mathrm{structure}}=2^{-\frac{1}{l}\sum i=1^{l}\log\;2P(\mathrm{residu}e_{i}|\mathrm{structur}e_{i})}$
	Quantifies amino acid compatibility with structural context (helix, sheet, buried, exposed). Estimated using AlphaFold confidence (pLDDT). *l* = protein length; $P(\mathrm{residu}e_{i}|\mathrm{structur}e_{i})$ = amino acid probability in context
Regulatory motifs	$PP_{\mathrm{regulatory}}=2^{-\frac{1}{m}\sum j1^{m}\log\;2P=(\mathrm{moti}f_{j}|\mathrm{regulome})}$
	Measures transcription factor binding site compatibility with recipient's regulatory architecture
	*m* = regulatory motifs; $P=(\mathrm{moti}f_{j}|\mathrm{regulome})$ = motif frequency in regulatory architecture
Extended regulatory	$PP_{\mathrm{extendedregulatory}}=P_{\mathrm{motif}}\times P_{\mathrm{temporal}}\times P_{\mathrm{environmental}}$
	Extends regulatory perplexity to temporal (cell cycle, developmental) and environmental (condition-specific) compatibility beyond motif recognition.
Metabolic integration	$PP_{\mathrm{metabolic}}=2^{-\log\;2P_{\mathrm{FBA}}(\mathrm{viability}|\mathrm{reactions})}$
	Estimates metabolic network disruption and toxic intermediate accumulation using flux balance analysis. $P_{\mathrm{FBA}}$ = predicted biomass/growth rate when gene's reactions are added
Protein interactions	$PP_{\mathrm{interaction}}=2^{-\log\;2P_{\mathrm{model}}(\mathrm{interaction}|\mathrm{sequences})}$
	Quantifies protein-protein interface compatibility using AlphaFold-Multimer (ipTM scores). High confidence = low perplexity. $P_{\mathrm{model}}$ from structural modeling
Spatial/chromosomal	$PP_{\mathrm{spatial}}=2^{-\frac{1}{k}\sum i=1^{k}\log\;2P(\mathrm{contac}t_{i}|\mathrm{position})}$
	Measures operon/chromatin architecture compatibility. *K* = chromosomal contacts; $P(\mathrm{contac}t_{i}|\mathrm{position})$ = expected contact frequency (Hi-C data)

Not all perplexity dimensions apply equally to all genetic changes. Gene gain (HGT, introgression, mutation) involves all dimensions; gene loss involves only systems-level dimensions (metabolic, interaction, regulatory) while simultaneously removing perplexity the lost gene itself contributed.

Perplexity is inherently context-dependent: the same genetic element will have different perplexity values in different genomic backgrounds.

Weighting coefficients should be calibrated using experimental fitness data from systematic gene transfer experiments in the focal organism rather than assuming universal constants.

Beyond simple codon usage, estimating these terms requires constructing computational models: genome-scale metabolic models for $PP_{\mathrm{metabolic}}$ protein structural modelling (AlphaFold-Multimer) for $PP_{\mathrm{interaction}}$, Hi-C data for $PP_{\mathrm{spatial}}$, and pangenome machine learning for $PP_{\mathrm{content}}$.

### Empirical support for the perplexity framework

The perplexity framework is supported by empirical evidence across multiple biological scales. Codon perplexity is the most tractable dimension. Organisms evolve synonymous codon preferences shaped by tRNA availability and translation efficiency (Sharp and Li 1987). *Escherichia coli* strongly prefers CTG for leucine (∼50% of leucine codons), while *Bacillus subtilis* uses CTG at half this frequency (Nakamura et al. 2000). A *B. subtilis* gene transferred to *E. coli* would have high codon perplexity, manifesting as reduced translation. Codon optimization increases heterologous protein expression 10- to 100-fold (Perlak et al. 1991), and imported genes' codon usage gradually matches hosts over time (Callens et al. 2021). Transformer-based genome models (DNABERT (Ji et al. 2021), Evo (Nguyen et al. 2024)) provide direct perplexity calculation by learning statistical patterns defining expected versus unexpected sequences.

Structural perplexity arises from proteins evolved amino acid compositions for structural contexts. Alpha helices prefer alanine, glutamate, and leucine, while beta sheets prefer valine, isoleucine, and tyrosine. A proline in an alpha helix creates high structural perplexity. AlphaFold (Evans et al. 2022; Jumper et al. 2021) encodes these perplexities through learned propensities, with low confidence predictions (pLDDT) indicating high structural perplexity.

Regulatory perplexity affects gene expression through transcription factor binding sites and promoter architecture that coevolve within genomes. Synthetic biology provides clear evidence: *E. coli* promoters (PBAD, PRha) depend on CRP but in *Pseudomonas aeruginosa* become dependent on Vfr, whose activity integrates into quorum-sensing and stress pathways, causing the “same” promoter to respond to entirely different signals (McMackin et al. 2021). This extends beyond motif recognition to temporal (cell cycle, developmental stage) and environmental compatibility.

Metabolic perplexity reflects tightly integrated metabolic networks where enzyme activities must balance to avoid toxic intermediates. Flux balance analysis (FBA) provides computational frameworks for estimation. A chimeric terpene synthase producing novel compound Y would have high metabolic perplexity if the recipient has no enzymes capable of metabolizing Y, but low perplexity if multiple pathways can use Y. Metabolic perplexity is environment-dependent: Tryptophan biosynthesis genes are beneficial in tryptophan-depleted environments but costly in tryptophan-rich ones.

Interaction perplexity arises from incompatible protein-protein interfaces, causing failed complex formation or dominant-negative effects. AlphaFold-Multimer interface confidence scores (ipTM) (Evans et al. 2022) enable quantitative estimation: High confidence indicates compatible interfaces (low perplexity), and low confidence suggests incompatibility.

Spatial perplexity affects genes in prokaryotic operons and eukaryotic chromatin domains. In eukaryotes, topologically associating domains (TADs) structure three-dimensional genome organization, although recent studies suggest substantial buffering and redundancy in TAD structure, limiting the impact of spatial rearrangements (Despang et al. 2019; Williamson et al. 2019). Genes acquired individually rather than as complete operons might end up with high spatial perplexity from the absence of co-regulated partners. This is particularly important for toxin-antitoxin systems like parD (Gerdes et al. 1990). Hi-C data quantifies expected chromatin contacts for transgene insertion prediction.

Content perplexity reflects gene presence–absence patterns in pangenomes. Beavan et al. (2024) showed in *E. coli* that the genes *pac* and *symE* exist at high frequency with high horizontal transfer rates, yet no genome contains both. In contrast, the presence of the gene *lgoT* predicts *mdtM* presence, but not vice versa. This is analogous to mutualistic gene relationships. Machine learning models trained on pangenome matrices can predict incompatible or synergistic gene combinations.

### Integration, predictions, and evolutionary implications

Perplexity is inherently context-dependent: The same genetic element has different perplexity values in different genomic backgrounds. A gene from a close relative may integrate with minimal perplexity, while the same gene in a distant species may have high perplexity across multiple dimensions. This explains why HGT success rates vary with phylogenetic distance and ecological context (Baltrus 2013; Hall et al. 2020; McInerney et al. 2020; Sela et al. 2021).

Total perplexity affecting fitness is a weighted sum across the various levels (see Table 1):


(1) $$\begin{array}{rl} PP_{\mathrm{total}}\;= & \alpha PP_{\mathrm{codon}}+\beta PP_{\mathrm{regulatory}}+\gamma PP_{\mathrm{metabolic}}+\delta PP_{\mathrm{interaction}}\\ & +\varepsilon PP_{\mathrm{structure}}+\zeta PP_{\mathrm{spatial}}+\theta PP_{\mathrm{content}} \end{array}$$

Weighting coefficients (α, β, γ, δ, ε, ζ, θ) are context-dependent and organism-specific, requiring empirical calibration using fitness measurements from systematic HGT experiments (Sorek et al. 2007). This is similar to how machine learning practitioners tune hyperparameters rather than relying on universal constants. Not all dimensions apply to all genetic changes: Gene gain involves all dimensions, whereas gene loss involves only systems-level dimensions (metabolic, interaction, regulatory) while removing perplexity the lost gene contributed.

Perplexity can indicate innovation potential in the sense that unexpectedness enables novel functions that the ancestral genome could not perform. If an unexpected element produces beneficial metabolic or regulatory novelty despite genomic incoherence, selection favors retention. Over time, integration through codon adaptation (Sharp and Li 1987), regulatory rewiring (Ochman et al. 2000), and compensatory mutations (Pál et al. 2006) decreases perplexity, and ultimately, the element becomes “expected” as fitness costs decline while benefits remain. Perplexity measurements can identify generalist elements (low perplexity in many hosts) and high-cost, high-reward elements (high retained perplexity).

The framework generates testable predictions. Retention probability balances functional benefit against perplexity cost:


(2) $$\mathrm{Retention}\propto \frac{\mathrm{Fitness}\;\mathrm{Benefit}}{\mathrm{Perplexity}\;\mathrm{Cost}}$$

First, retained acquired elements should show perplexity reduction, perhaps in the form of codon adaptation, regulatory element evolution, or integration into endogenous networks. Second, genes with sustained high perplexity must provide substantial selective benefit justifying retention costs. Third, genes duplicating existing functions are lost rapidly regardless of low perplexity (Sorek et al. 2007). Conversely, novel metabolic pathways may persist despite initial high perplexity if selective benefit outweighs integration costs. Failed acquisition events can be identified through truncated sequences retaining high-perplexity markers: divergent codon usage, poorly recognized regulatory elements, and lack of endogenous network integration. High-perplexity genomic regions that persist represent genes that survived despite genomic incoherence. Tolerance for high-perplexity transfers may vary. Organisms with flexible, redundant architectures may accommodate HGT more readily than those with tightly integrated genomes, potentially explaining variation in mutation-permissiveness across taxa (Ogier et al. 2010). At the extreme, this tolerance may explain why whole-genome duplication, which would create simultaneous perplexity across all molecular dimensions, has occurred repeatedly in eukaryotes, including plants (Jiao et al. 2011), animals (Dehal and Boore 2005), and fungi (Wolfe and Shields 1997). This demonstrates that some lineages possess sufficient architectural flexibility to survive massive genomic upheaval.

## Empirical evidence for context-dependent fitness effects

The fact that gene function is context-dependent is well-known in many fields of study, including developmental biology and population genetics. Pangenomes have demonstrated these effects across thousands of genomic contexts simultaneously. Pangenomic studies have demonstrated context-dependent gene essentiality (Rosconi et al. 2022), identifying genes that are essential for viability in one organism while being dispensable in closely related strains, showing that even the lethality phenotype depends on genomic context. Orthologous genes in different *S. pneumoniae* strains have manifested different essentiality profiles despite their genomic and metabolic similarity (Beavan and McInerney 2022; Rosconi et al. 2022). Consistently essential genes tend to be more highly conserved across evolutionary history (Bao et al. 2025), suggesting that selection acts on the essentiality of a gene in each genomic context. Essentiality, therefore, is not an intrinsic property of the gene itself; rather, it emerges from the relationship between the gene and its genomic environment. There are no more than about 31 universally conserved genes across all cellular life (Ciccarelli et al. 2006), and if we include viruses and phage, then there are none. A single gene's essentiality profile, or its fitness effect profile across a pangenome, is therefore a map of how that gene's function depends on genomic context.

To operationalize the quantitative concept of genomic perplexity, we must demonstrate how the weighted sum of its various dimensions (Table 1 and Equation 1) applies across the full spectrum of evolutionary events. Table 2 illustrates, using empirical studies, the costs and potential benefits associated with various genetic changes, ranging from single synonymous substitutions (codon perplexity) to large-scale events like HGT and chromosomal inversions (spatial perplexity). These studies, and others, demonstrate the framework's universality, highlighting that every kind of genomic change has the potential to induce a measurable cost tied to statistical unexpectedness. The detailed costs and benefits reinforce the principle that long-term gene retention is governed by the balance between functional benefit and the systematic cost imposed by perplexity (Equation 2). Furthermore, these studies emphasize that the functional outcome of any genetic change is inherently context-dependent, showing how the same event can be deleterious or beneficial based on the organism's specific genomic, metabolic, or environmental background.

**Table 2:** Genomic perplexity creates both fitness costs and innovation potential.

Genetic change	Perplexity source	Potential fitness cost	Potential benefit	Context determining outcome	Citation
Synonymous substitution	Codon usage bias	Reduction in translation rate; ribosome stalling	Fine-tuning of expression level; translational regulation under stress	Usually costly; beneficial when precise expression control needed	Hershberg and Petrov (2008); Kudla et al. (2009); Plotkin and Kudla (2011); Sørensen and Pedersen (1991)
Nonsynonymous mutation (structural)	Structural context	+ve ΔΔ*G* values; protein aggregation	Altered stability enables function at different temperatures or pH	Deleterious in native environment; adaptive in new thermal/pH regime	Bershtein et al. (2008); Bloom et al. (2010); DePristo et al. (2005); Tokuriki and Tawfik (2009); Tokuriki et al. (2007)
Nonsynonymous mutation (interaction)	Interaction context	Reduction in native binding affinity	Gain of novel binding partner; neo-functionalization	Loss of ancestral function; gain of new function (trade-off)	Ashenberg et al. (2013); Bloom et al. (2006); Gong et al. (2013); Starr et al. (2021); Starr et al. (2022)
Regulatory mutation	Transcription factor context	Mistimed expression; fitness cost in wrong conditions	Gain of condition-specific expression; stress response evolution	Costly in constant environment; beneficial when conditions fluctuate	Berg et al. (2004); Friedlander et al. (2016); Lynch and Hagner (2015); Mustonen and Lässig (2005); Wittkopp and Kalay (2011)
Gene duplication	Stoichiometric balance	Toxic imbalance; growth defect from dosage imbalance	Redundancy enables subfunctionalization; dosage-dependent benefits	Initially costly; long-term substrate for innovation	Birchler and Veitia (2012); Papp et al. (2003); Teufel et al. (2016); Veitia et al. (2008)
Promoter swap	Expression level context	Growth rate reduction; metabolic burden	Escape from native regulation; novel expression patterns	Costly with constitutive expression; beneficial with inducible control	Alper et al. (2005); McNally et al. (2016); Poirel et al. (2005); Porse et al. (2016)
Domain insertion/shuffling	Interface compatibility	Nonfunctional chimera	Novel substrate specificity; new enzymatic activity	Most fail; rare successes create major innovations	Lehmann et al. (2002); Romero and Arnold (2009); Voigt et al. (2002)
Operon disruption	Co-regulation context	Pathway imbalance; auxotrophy from broken operons	Breaking co-regulation allows independent optimization	Deleterious for tightly coupled pathways; beneficial for flexible regulation	Lawrence and Roth (1996); Price et al. (2005); Price et al. (2006)
Chromosomal inversion	Spatial organization context	Disrupts TADs and replication timing	Brings distant enhancers to new genes; creates local adaptation	Costly in lab; can be strongly adaptive in nature (seasonality)	Ayala et al. (2013); Berdan et al. (2021); Corbett-Detig and Hartl (2012); Wellenreuther and Bernatchez (2018)
HGT: codon usage	Codon bias mismatch	Reduction in expression; slow translation	Novel gene with adaptive function can persist despite cost	Ameliorates over ∼10^4^ to 10^6^ generations if function beneficial.	Lawrence and Ochman (1997); Medrano-Soto et al. (2004); Navarre et al. (2006); Tuller et al. (2010); Tuller et al. (2011)
HGT: regulatory	Promoter incompatibility	Silent gene; no expression without native σ-factor	Escapes host regulation; expression in novel conditions	Remains silent until compensatory mutations; then conditionally expressed	Dorman (2007); Lucchini et al. (2006); Navarre et al. (2007); Shintani et al. (2015)
HGT: metabolic	Pathway integration	Metabolic burden without substrate	Novel carbon source utilization; antibiotic synthesis	Negative selection in absence of substrate; strong positive selection when present	Cordero and Hogeweg (2009); Lercher and Pál (2008); Pál et al. (2005); Price et al. (2008)
HGT: protein interaction	Interaction incompatibility	Lethal if toxin acquired without antitoxin	Complete functional system (eg restriction-modification and CRISPR)	Barrier prevents most transfer; co-transfer of system enables innovation	Jørgensen et al. (2009); Makarova et al. (2009); Van Melderen and Saavedra De Bast (2009)
HGT: combined effects	All perplexity sources simultaneously	Initial fitness cost across multiple levels	Radical functional innovation; access to entirely new niches	Only 1% to 10% of transfers fixed; retained ones show high benefit/cost ratio	Baltrus (2013); Frost et al. (2005); Gogarten and Townsend (2005); Soucy et al. (2015)

Genes acquired horizontally from distantly related species often confer reduced fitness initially (Baltrus 2013), not because they are inherently defective but because they arrive in a genomic context optimized for a different set of genetic components than the donor species. Over time, either the transferred genes adjust through mutation, or the recipient genome undergoes compensatory changes, until the new genes become integrated into a functional whole (Yang et al. 2020).

Synthetic biological circuits provide instructive examples of context-dependency. Carefully designed genetic circuits that function predictably in laboratory strains often fail or behave unpredictably when transferred to closely related hosts (Cardinale and Arkin 2012; Stone et al. 2024). Promoter and repressor combinations that drive strong expression in *E. coli* may be weak or inactive in other species, such as *Pseudomonas putida* (Tas et al. 2021). Metabolic pathways engineered in the yeast *Saccharomyces cerevisiae* can produce unexpected regulatory bottlenecks or metabolic imbalances when introduced into the yeast *Candida albicans*, for example (Ostergaard et al. 2000). These failures are not failures of the DNA sequence itself but rather highlight features that must be accounted for, to understand how the sequence functions within different genomic and metabolic contexts.

Pleiotropic mutations exemplify context-dependency: The same mutation causes intellectual disability, pigmentation defects, and immune dysfunction depending on when and where the gene is expressed (Menasche et al. 2000), with a transcription factor essential in developing brain being silent in muscle or a metabolic enzyme playing different roles depending on nutritional state. Rather than a single gene product having multiple functions, this illustrates how the same gene produces different functional outcomes across cellular, developmental, and physiological contexts.

Context-dependency is also seen in the persistent gap between genotype and phenotype prediction. Genome-wide association studies (GWAS) identify variants associated with disease risk, with identical variants showing variable penetrance across populations, families, and even individuals. A variant that doubles disease risk on average in 1 genetic background may have minimal effect in another (Sinnott-Armstrong et al. 2024). This emphasizes the point that gene effects are not universal properties but emerge from specific genomic and environmental contexts. Machine learning approaches might work better to close this knowledge gap: They can predict phenotypes from data without needing to understand the underlying biology. Recognizing patterns can sometimes prove superior to understanding mechanisms, when systems are too complex to understand mechanistically (Breiman 2001; Jumper et al. 2021).

## Selection on probability distributions

The context-dependent framework predicts that the focus of natural selection is not on a fixed gene activity but on probability distributions of functional outcomes across contexts. In language models, attention mechanisms determine how strongly different elements of context influence outcomes. Biological systems exhibit analogous patterns, which can be seen at multiple levels. Epistatic interactions determine how strongly genetic loci influence each other's effects, gene-by-environment interactions determine how environmental factors modulate gene function (Baier et al. 2023), and structural constraints determine how molecular components influence protein function. These biological attention patterns are not learned through training but have evolved through natural selection, creating the context-dependent functional outcomes we observe.

When I refer to attention in biological systems, I use the term as a conceptual analogy rather than a mechanistic equivalence. I mean the degree to which one element's state influences another element's state, and together they determine the functional outcome. A genetic locus that strongly modulates another gene's effect can be said to confer high epistatic attention to that gene. An environmental factor that strongly selects for a protein's function has high environmental attention. A residue whose state determines protein activity has high structural attention. Unlike machine learning, where attention weights are optimized during training, biological attention patterns emerge through the standard evolutionary processes of mutation, selection, and drift that together shape how elements influence each other. Future developments in biologically plausible transformer architectures trained on multiomics data may eventually enable more direct mapping between computational attention and biological context-weighting mechanisms.

If genes function through context-dependent probability distributions rather than deterministic mappings, what does natural selection act on? Selection favors genomes that produce adaptive phenotypes with appropriate probability distributions across the contexts that organisms encounter. A genome producing identical phenotypes regardless of condition would be brittle; one producing random phenotypes would be chaotic. Evolution selects for genomes that generate modal phenotypes for typical conditions while retaining capacity for diverse responses when conditions are unusual (bet-hedging) and rapid probability shifts in response to environmental cues (plasticity). This is achieved through regulatory flexibility, alternative splicing, stochastic expression, and over longer timescales, recombination, and HGT.

The perplexity framework generates quantitative predictions for fundamental patterns in pangenome evolution. Natural selection acts on the frequency-weighted expected fitness across realized contexts:


(3) $$\bar{W}(g)=\int f(g,c)\cdot p(c)dc$$

where $\bar{W}(g)$ is the mean fitness of genotype *g*, f(g,c) is the fitness of genotype g in context *c*, and p(c) is the probability of encountering context *c*. Here f(g,c) doesn’t represent variation in biochemical activity, but variation in whether that activity contributes to survival and reproduction in context *c*. A mutation or horizontally transferred gene is favored when it increases this expectation:


(4) $$\Delta \bar{W}=\bar{W}(g^{\prime })-\bar{W}(g)>0$$

where *g*’ is the genotype with the new mutation or horizontally transferred gene, $\bar{W}(g)$ is its mean fitness calculated as in Equation 3, and $\Delta \bar{W}$ is the change in mean fitness.

Natural selection samples this distribution through successive generations. Perplexity quantifies how genomic incompatibility (through codon usage, regulatory logic, protein interactions, and other molecular factors) creates systematic fitness costs that both shift the fitness distribution and favor compensatory changes that reduce incompatibility.

This framework can provide a conceptual explanation for the U-shaped gene frequency distribution that is characteristic of most prokaryotic pangenomes (Domingo-Sananes and McInerney 2021; McInerney et al. 2017). Core genes that are present in all strains should exhibit low perplexity across diverse genomic contexts (ie core genes typically have optimized synonymous codon usage that matches the most abundant cognate tRNAs in the cytoplasm (Ikemura 1985; Sharp and Li 1987)), maintaining high $\bar{W}$ through strong purifying selection on their presence. Rare accessory genes at the opposite end of the frequency spectrum are expected to show high $PP_{\mathrm{total}}$ in most contexts, but they persist because they provide substantial context-specific benefits where $f(g,c^{\prime })$ is strongly positive despite general incompatibility. The relative absence of intermediate-frequency genes also emerges naturally in this framework. Genes with moderate perplexity and weak benefits are eliminated by selection, while those at intermediate frequencies are either recently acquired genes undergoing perplexity reduction through amelioration, or genes maintained by frequency-dependent selection or balancing selection across contexts (Harrow et al. 2021).

The framework further explains variation in pangenome openness across species. Open pangenomes characteristic of cosmopolitan species result from the interplay of large *N_e_* and high environmental variability. Species like *E. coli* maintain strong codon biases (high α in $PP_{\mathrm{total}}$), tightly integrated metabolism (high γ), and specific regulatory requirements (high β), creating substantial perplexity costs for foreign genes. However, their large *N_e_* enables natural selection to detect small fitness advantages, while high environmental variability (large Var(p(c))) creates numerous contexts where accessory genes provide substantial benefits. This tips the balance in favor of an open pangenome (Beavan et al. 2024; Cummins et al. 2022; Dillon et al. 2025; Hall et al. 2021):


(5) $$\mathrm{Openness}\propto \frac{N_{e}\times \mathrm{Var}(\;p(c))}{\sum_{i}\;w_{i}\cdot PP_{i}}$$

where $w_{i}$ represents the weights (α, β, γ, δ, ε, ζ, θ) and the denominator is the weighted total perplexity cost. Conversely, closed pangenomes in specialist species like *Mycobacterium tuberculosis* experience low environmental variability (small Var(p(c)))) and often smaller *N_e_*. So, although relaxed genomic constraints (lower $w_{i}$ values) will reduce perplexity costs, the limited contexts where accessory genes provide benefits result in closed pangenomes (Behruznia et al. 2025).

The distribution of fitness effects (DFE) for new mutations and horizontally transferred genes emerges directly from how perplexity reshapes fitness across contexts. Most new genes exhibit negative $\Delta \bar{W}$ because high perplexity across multiple dimensions ($PP_{\mathrm{codon}}$, $PP_{\mathrm{regulatory}}$, $PP_{\mathrm{metabolic}}$, and so forth) systematically reduces f(g,c) across most contexts, creating a heavy left tail in the DFE. Beneficial mutations occupy the right tail. These are genes whose novel function in specific contexts outweighs integration costs. Importantly, the DFE is not static but context-dependent. A gene that is beneficial in one genomic background (low perplexity, needed function) might prove deleterious in another (high perplexity, redundant function). The DFE becomes more positive-skewed during environmental change, niche expansion, or antibiotic exposure and more negative during genome streamlining in stable environments.

Effective population size ($N_{e}$) determines which perplexity costs are visible to selection, through drift-selection balance (Sung et al. 2012). Natural selection operates effectively when:


(6) $$|\Delta \bar{W}|\gg \frac{1}{2N_{e}}$$

where


(7) $$\Delta \bar{W}\approx \mathrm{Benefit}(c)-\mathrm{Cost}(PP_{\mathrm{total}})$$

In large populations, selection detects even small fitness differences, maintaining accessory genes with modest context-specific advantages. Recent work confirms that accessory genes are, on average, slightly beneficial (Douglas and Shapiro 2024). This enables accumulation of specialized genes producing the large, open pangenomes of cosmopolitan species like *E. coli*.

In small populations, only genes with substantial net benefits overcome drift, producing smaller, closed pangenomes. This is seen most clearly in specialized organisms and endosymbionts (McInerney et al. 2017). Between these extremes lies an effectively neutral space where:


(8) $$|\mathrm{Benefit}-PP_{\mathrm{total}}|<\frac{1}{2N_{e}}$$

Gene frequencies follow:


(9) $$p(\mathrm{frequency}|g)\propto e^{4N_{e}\cdot \Delta \bar{W}\cdot f\cdot (1-f)}$$

This produces 3 regimes governing gene frequency trajectories. $\Delta \bar{W}\gg \frac{1}{2N_{e}}$ increases frequency, $|\Delta \bar{W}|\approx \frac{1}{2N_{e}}$ allows random frequency wandering, and weak negative selection, while ($\Delta \bar{W}<-\frac{1}{2N_{e}}$) maintains genes at low frequency or results in their loss from the population. These regimes are context-dependent, and $\Delta \bar{W}$ shifts as the genomic background evolves (through mutations, recombination, or other HGT events) and as environmental conditions change. A gene favored in the current context may face purifying selection after compensatory mutations alter the genetic background or when the organism encounters different environments, preventing stable fixation and generating the dynamic frequency distributions and intermediate-frequency genes characteristic of open pangenomes.

Together, these relationships suggest that measurable molecular features such as synonymous codon usage, regulatory element compatibility, metabolic network integration, protein interaction interfaces, structural propensities, chromosomal organization, and gene co-occurrence patterns may quantitatively predict evolutionary outcomes across scales from individual mutations to pangenome architecture. The perplexity framework provides a unified, testable explanation for the distribution of gene frequencies, the dichotomy between open and closed pangenomes, the shape of the fitness distribution, and the modulating role of population size and genetic drift.

Natural selection acts on genotypes by differentially propagating those that generate fitness distributions with higher frequency-weighted expectations across the contexts that are encountered during evolutionary history. The fate of any genetic change depends not on its effect in any single context, but on how it reshapes the entire distribution of outcomes. In other words, how it shifts the mean of the distribution, its variances, and how it changes its tail probabilities. These changes are all integrated across the contexts weighted by the frequency with which the contexts are encountered. Every genome is therefore a compressed representation of these distributions.

The extreme genetic variation in prokaryotic pangenomes (McInerney et al. 2017) becomes expected under this framework. A new variant that shifts phenotypic distributions toward beneficial outcomes in one context may be neutral or harmful in another (Domingo-Sananes and McInerney 2021). While external environments and genomic backgrounds fluctuate, selection maintains multiple alleles because different variants are beneficial under different conditions. Polymorphisms in regulatory elements might reduce expression variance in stable conditions but enable bet-hedging when conditions fluctuate (Metzger et al. 2015; Veening et al. 2008), maintaining intermediate frequencies depending on environmental fluctuation rates (Clarke and O'Donald 1964; Harrow et al. 2021). We do not need to know the distribution's shape, dimensionality, or moments, because selection samples it through survival and reproduction across generations. Context-dependency is therefore not a complication but central to evolutionary dynamics.

From the perspective of a deterministic model, epistasis would appear only exceptionally. Most genes would work independently, with occasional interactions. Under context-dependent models, epistasis is universal because gene effects always depend on genetic background. The question is not whether epistasis exists but how strong it is. Strong epistasis (eg synthetic lethality) is noteworthy, but weak epistasis, where a gene's effect depends slightly on genetic background, is universal and expected (Phillips 2008). Evolutionary models treating genes as independent loci systematically underestimate unpredictability, while models embracing epistasis as foundational become more realistic, although computationally complex.

Context-dependent gene function enables both robustness and evolvability. Redundancy and regulatory flexibility provide multiple genetic paths to the same outcome (robustness), while this same flexibility enables small changes in genetic background or environment to shift which phenotypic response is produced (evolvability). Selection favors genomic architectures generating adaptive probability distributions across conditions through regulatory modularity (Melo and Marroig 2015), genetic redundancy (Dillon et al. 2025), and stochastic expression that generates phenotypic diversity within clonal populations (Barnett et al. 2025).

## Conclusion

Context dependence is fundamental to evolution. The outcome of any mutation is conditioned on genetic background, environment, and demographic history. The perplexity framework makes this quantitative, generating testable predictions for gene frequency distributions, HGT success rates, and the relationship between environmental variability and gene, genome, and pangenome structure. Language models provide both the mathematical tools (attention, probability distributions, perplexity) and a computational parallel. Genomes and neural networks both encode flexible, context-responsive function in learned statistical patterns.

This perspective addresses long-standing tensions, including why genetic variation persists, why synthetic circuits fail upon transfer, why genotype–phenotype prediction remains difficult, and why horizontally transferred genes face fitness costs but still they frequently integrate. Instead of asking “What does this gene do?”, we should ask “What can this gene become?” This requires treating genomic context as a primary object of study and systematically testing genes across multiple genomic backgrounds rather than just comparisons of knockout versus wild-type. Practical applications of genomics, from synthetic biology to antimicrobial development, become questions of context engineering rather than gene optimization alone.

This raises a subtle but important point about molecular evolution. When we observe strong conservation at particular amino acid positions or we see positive selection on specific codons (Yang and Nielsen 2002), we are not documenting selection on the position itself but on its contribution to system-level functional outcomes across contexts. The conservation pattern is real and is caused by natural selection, but selection for integrated function across contexts, not for fixed, position-specific properties. Identifying conserved and variable sites is still very much of deep importance, but understanding that natural selection is acting on probability distributions across contexts enriches our understanding of why they’re conserved. While prokaryotic pangenomes provide the clearest empirical ground for observing context-dependent function, the same dynamics underlie all evolutionary systems, from gene networks to neural circuits, in which information interacts with the environment to produce phenotype. This is a perspective aligned with broader conceptualizations of contextual organismality (Diaz-Munoz et al. 2016) and recent work on unifying principles across evolutionary systems (Tingle 2025).

Extending machine learning architectures to large-scale multiomics datasets would enable direct calculation of context-dependent fitness predictions. Rather than training separate models per genome, a pangenome transformer could learn which genetic contexts prefer which accessory genes, quantifying the full $PP_{total}$ across all molecular dimensions simultaneously. This approach could predict gene transfer success, identify compensatory mutation pathways, and explain strain-specific phenotypic variation, thereby making the perplexity framework operationally testable at the genomic scale.

The impossibility of exhaustive functional cataloging is precisely why this probabilistic framework is necessary. Rather than attempting to document every possible gene function across all contexts, I propose studying the principles that govern how genomic context shapes functional probability distributions. This shift would mimic how natural language processing succeeded not by encoding exhaustive word definitions and grammatical rules but by learning statistical patterns from language use. Modern language models make accurate predictions about word meanings and sentence structure without explicitly cataloging every possible usage. Instead, they characterize the distributions that emerge from linguistic context. Similarly, we can make testable predictions about gene function, HGT success, and evolutionary trajectories by characterizing the statistical properties of genomic contexts rather than enumerating all possible outcomes.

## Data Availability

There is no data associated with this manuscript.
